# Abbott’s at the Top

**Published:** 1909-10

**Authors:** 


Abbott's at the Top.—The attention of our readers is called to the striking full-page advertisement of The Abbott Alkaloidal Company on page —. The idea is particularly appropriate at this time. Abbott's Saline Laxative occupies a high place in the esteem of the medical profession. Dr. Abbott's "Clean Out, Clean Up and Keep Clean" slogan has gained ground so rapidly that the output of his effervescent salines is larger than ever before—increasing by leaps and bounds. Liberal samples will be sent to interested physicians mentioning this journal.

